# Simultaneous Determination of Multi-Mycotoxins in Cereal Grains Collected from South Korea by LC/MS/MS

**DOI:** 10.3390/toxins9030106

**Published:** 2017-03-16

**Authors:** Dong-Ho Kim, Sung-Yong Hong, Jea Woo Kang, Sung Min Cho, Kyu Ri Lee, Tae Kyung An, Chan Lee, Soo Hyun Chung

**Affiliations:** 1 National Agricultural Products Quality Management Service, 5-3 Gimcheon innocity, Nam-myeon, Gimcheon City Gyeongsangbuk-do Province 39660, Korea; anoldmu@korea.kr; 2 Department of Integrated Biomedical and Life Science, Korea University, Seoul 02841, Korea; lunohong@korea.ac.kr (S.-Y.H.); wodn5000@gmail.com (J.W.K.); gangnangie@korea.ac.kr (S.M.C.); leekr0511@naver.com (K.R.L.); 3 Department of Food Science and Technology, Chung-Ang University, Anseong-si, Gyeonggi-do 17546, Korea; cobyatk@naver.com

**Keywords:** aflatoxins, deoxynivalenol, fumonisins, HT-2, nivalenol, ochratoxin A, T-2, zearalenone, grains, LC/MS/MS

## Abstract

An improved analytical method compared with conventional ones was developed for simultaneous determination of 13 mycotoxins (deoxynivalenol, nivalenol, 3-acetylnivalenol, aflatoxin B_1_, aflatoxin B_2_, aflatoxin G_1_, aflatoxin G_2_, fumonisin B_1_, fumonisin B_2_, T-2, HT-2, zearalenone, and ochratoxin A) in cereal grains by liquid chromatography-tandem mass spectrometry (LC/MS/MS) after a single immunoaffinity column clean-up. The method showed a good linearity, sensitivity, specificity, and accuracy in mycotoxin determination by LC/MS/MS. The levels of 13 mycotoxins in 5 types of commercial grains (brown rice, maize, millet, sorghum, and mixed cereal) from South Korea were determined in a total of 507 cereal grains. Mycotoxins produced from *Fusarium* sp. (fumonisins, deoxynivalenol, nivalenol, and zearalenone) were more frequently (more than 5%) and concurrently detected in all cereal grains along with higher mean levels (4.3–161.0 ng/g) in positive samples than other toxins such as aflatoxins and ochratoxin A (less than 9% and below 5.2 ng/g in positive samples) from other fungal species.

## 1. Introduction

Mycotoxins are biologically active secondary metabolites produced by a variety of fungi such as *Aspergillus*, *Penicillium*, and *Fusarium* sp. To date, approximately 400 compounds have been identified as mycotoxins such as aflatoxins (AFs), ochratoxin A (OTA), fumonisins (Fs), nivalenol (NIV), deoxynivalenol (DON), zearalenone (ZEN), T-2 toxin (T-2), and HT-2 toxin (HT-2) [1]. The International Agency for Research on Cancer [2] assigned major mycotoxins into one of 5 groups based on their carcinogenicity. Aflatoxin B_1_ (AFB_1_) is categorized as a Group 1 human carcinogen due to its potent carcinogenic properties in liver [2]. OTA, fumonisin B_1_ (FB_1_)_,_ and fumonisin B_2_ (FB_2_) are classified as possible carcinogens (Group 2B) to human since OTA causes nephrotoxicity, immune suppression, carcinogenicity, and teratogenicity in laboratory animals, and it has been associated with a fatal human kidney disease known as Balkan Endemic Nephropathy, and FB_1_ and FB_2_ cause equine leukoencephalomalacia (ELEM) in horses and porcine pulmonary edema (PPE) in pigs [3]. However, DON, ZEN, and T-2 are categorized into Group 3 (not classifiable as to its carcinogenicity to humans) because there is no evidence in their mutagenicity and carcinogenicity [4].

These mycotoxins occur in agricultural crops during pre-harvest and storage. As the mycotoxins are chemically very stable, they are not degraded during food processing, causing a variety of adverse and toxic health effects in target organs such as liver, kidney, and nerve systems in human. Thus, most of the countries started to reinforce management of mycotoxins in foods and feeds, and the European Union and the Codex have made efforts to set common regulatory limits for mycotoxins. South Korea has also set and regulated the maximum allowable limits for important mycotoxins in foods and feeds based on Food Sanitation Act and Control of Livestock and Fish Feed Act. Currently, risks of mycotoxins found in agricultural crops are assessed by analyses of only one toxin among several mycotoxins, which can contaminate the same agricultural crops, and the maximum allowable limits and analytical methods for major mycotoxins are established only for individual toxin in South Korea as well as other countries. Moreover, these mycotoxins found in a contaminated agricultural commodity can cause serious synergistic effects in human and animals when consumed simultaneously by them. Boeira and co-workers reported a synergistic toxicity between *Fusarium* mycotoxins (DON and ZEA) on the growth of brewing yeasts [5]. Other researchers have demonstrated that a combined toxicity of either DON and Fs or AFs and FB_1_ in liver in piglets or barrows caused higher histopathological lesion and immune suppression [6,7]. Severe reductions in growth and immune response were found in broilers by dietary combinations of AFs and OTA [8]. Also, synergistic cytotoxic effects of mycotoxin combinations were shown in mammalian cell [9,10,11,12]. Because co-occurrence of mycotoxins is very common in agricultural commodities, a reliable and sensitive analytical method is needed for simultaneous determination of multi-mycotoxins. In addition, a prerequisite for the analyses of multi-toxins is good recoveries of the toxins at toxin extraction and clean-up steps. Most of recently developed multi-mycotoxin analytical methods employ acetonitrile-water mixtures for co-extraction of mycotoxins at toxin extraction steps [13]. In order to purify toxin extracts at toxin clean-up steps, commercial immunoaffinity columns (IAC) have been successfully applied for simultaneous determination of mycotoxins by liquid chromatography-tandem mass spectrometry (LC/MS/MS). There are two toxin elution methods at IAC steps; single elution [13] and double elution [14]. In the current study, in order to develop analytical methods for rapid and efficient determination of major mycotoxins in food, we developed a new rapid method to co-elute all 13 major mycotoxins by one step using 5 mL of 80% methanol (MeOH) containing 0.5% acetic acid at the same time and established an analytical method for simultaneous determination of the 13 mycotoxins by LC/MS/MS. The method was successfully applied for rapid and simultaneous determination of the 13 mycotoxins in grains collected from retail markets in South Korea. To the best of our knowledge, this is the first report on improved co-extraction of 13 mycotoxins in a variety of grains and simultaneous analyses of the multi-mycotoxins in the cereal grains collected from markets in South Korea by LC/MS/MS.

## 2. Results

### 2.1. Linearity of Calibration Curves for 13 Mycotoxins 

**Figure 1:**
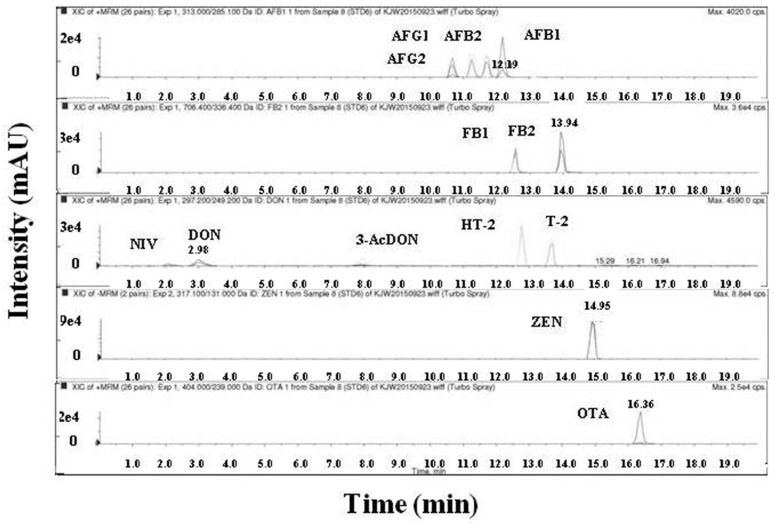
Chromatograms of 13 mycotoxins using LC/MS/MS. Extract ion chromatogram of 13 mycotoxins. Ten microliters of the toxin mixture standard was injected into the LC/MS/MS system. The retention times of peaks corresponding to each toxin are as follow: NIV, 2.08 min; DON, 2.98 min; 3-AcDON, 7.95 min; AFG_2_, 10.67 min; AFG_1_, 11.25 min; AFB_2_, 11.71 min; AFB_1_, 12.19 min; FB_1_, 12.54 min; HT-2, 12.77 min; T-2, 13.68 min; FB_2_, 13.94 min; ZEN, 14.95 min; OTA, 16.36 min.

The linearity of 13 mycotoxins in the analytical method was assessed by each standard curve using 5 levels of standard solutions for each toxin. An extract ion chromatogram (EIC) of 13 mycotoxins analyzed by the LC/MS/MS is shown in Figure 1. The linearity of the calibration curves was determined by linear regression analysis. The curves for all 13 mycotoxins showed *r^2^* values of 0.9932–1.0000 (Figure S1). Therefore, we concluded that the standard curves for all 13 mycotoxins were linear in the range of 1.3–53 ng/mL.

### 2.2. Extraction of 13 Mycotoxins from 5 Different Matrices and the Effects of the Matrices on Toxin Extraction

Previously, toxins have been extracted by two steps using water and MeOH, which require time-consuming and laborious shaking and clean-up [14,15]. In this study our method describes a rapid and efficient co-extraction and co-elution of 13 mycotoxins in 5 different matrices including mixed cereal with organic solvents containing acetic acid. Our one step method for extraction of multi-toxins shortened the toxin extraction procedure.

Extraction of toxins from grain samples with organic solvents entails the possibility of analytical problems (matrix effects) due to the co-extraction of matrix components in the samples. The matrix effects can affect the ionization efficiency of toxins, leading to suppression or enhancement of the signal in LC/MS/MS depending on combinations of types of toxins and matrices. Thus, the effects of matrices on the determination of 13 mycotoxins in 5 types of grains were evaluated.

The signal suppression/enhancement was calculated by the following equation:

$$SSE=\frac{Slope\;of\;a\;standard\;curve\;using\;a\;sample\;spiked\;with\;a\;toxin\;\times \;100}{Slope\;of\;a\;standard\;curve\;using\;a\;toxin\;standard\;solution}$$

**Figure 2:**
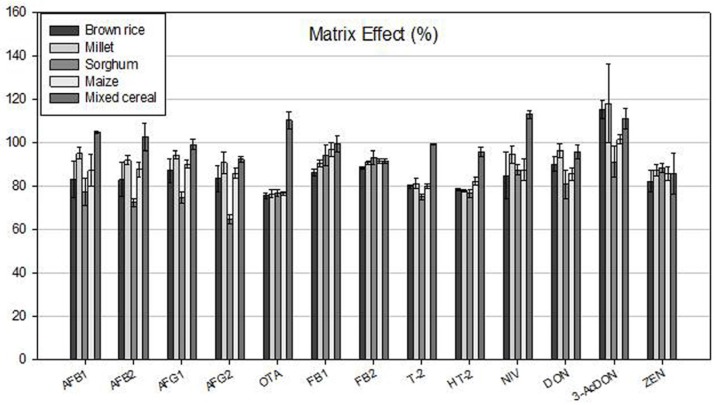
Signal suppression/ enhancement (SSE) effect of 13 mycotoxins in 5 types of matrices. Five levels (1.325, 2.65, 13.25, 26.5, and 53 ng/mL) of standard solutions for each toxin were prepared by mixing the extract from each type of grain sample. The standard solutions for each toxin were injected into LC/MS/MS in triplicate.

Five types of grain samples showed minor matrix effects on the determination of all 13 toxins in the samples (74.5%–112.2%) (Figure 2). Of these toxins, signals for levels of 3-AcDON in all 5 types of samples were slightly enhanced by the matrices (102.9%–112.2%), whereas those for all other toxins in the samples were a little suppressed by the matrices (74.5%–90.0%).

### 2.3. Recovery of 13 Mycotoxins from 5 Different Matrices

Recoveries for 13 mycotoxins extracted from each matrix spiked with each toxin standard solution were evaluated by using our newly developed co-extraction and co-elution method. 

The recoveries were calculated by the following equation:

$$\mathrm{Recovery}=\frac{\mathrm{Each}\;\mathrm{toxin}\;\mathrm{concentration}\;\mathrm{equivalent}\;\mathrm{to}\;\mathrm{the}\;\mathrm{peak}\;\mathrm{area}\;\mathrm{measured}\;\mathrm{from}\;\mathrm{the}\;\mathrm{spiked}\;\mathrm{sample}\times 100}{\mathrm{Each}\;\mathrm{toxin}\;\mathrm{concentration}\;\mathrm{used}\;\mathrm{for}\;\mathrm{spiking}\;\mathrm{the}\;\mathrm{sample}}$$

**Figure 3:**
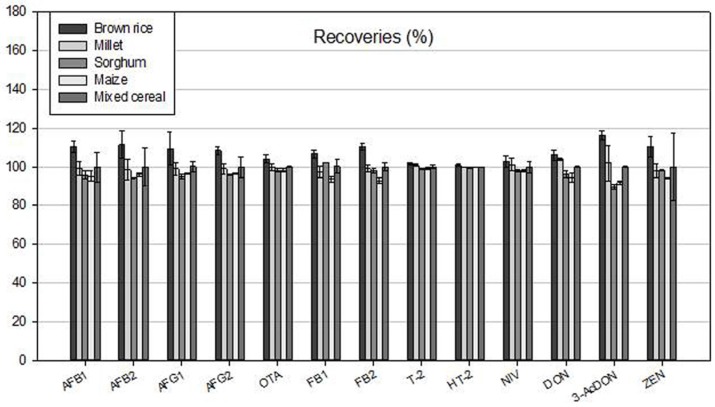
Recovery of 13 mycotoxins from 5 types of matrices. Each matrix, which was naturally uncontaminated with 13 mycotoxins, was spiked with 1.2–326.1 ng/mL of each toxin standard solution as described in materials and methods. Toxins extracted from each matrix were injected into LC/MS/MS.

The recoveries were measured by injecting toxins extracted from each matrix, which was naturally uncontaminated with 13 mycotoxins and spiked with 1.2–326.1 ng/mL of each toxin standard solution as described in materials and methods, into LC/MS/MS. The recovery rates for 13 mycotoxins in 5 types of matrices were 73.9%–133.0% along with relative standard deviation (RSD_r_) of 0.1%–14.3% (Figure 3). These recovery rates of 13 mycotoxins satisfied allowable limits of the recovery and RSD recommended by Codex or Association of Official Analytical Chemists (AOAC) [16,17]. The Codex recommends 60%–120% of recovery rates in food samples contaminated with 1–10 μg/kg of mycotoxins and the guideline for the recoveries by AOAC is 70%–125% in food samples contaminated with 10 μg/kg of mycotoxins. In addition, RSD_r_ values of the 13 mycotoxins (0.1%–14.3%) were below 15%, which is recommended for food samples contaminated with 10 μg/kg of mycotoxins by AOAC. Thus, we concluded that the analytical method by co-extraction and co-elution using MeOH containing acetic acid had good recoveries from 5 types of matrices.

### 2.4. LOD and LOQ of an Analytical Method for Determination of Levels of 13 Mycotoxins

The sensitivity of the analytical method using LC/MS/MS was determined by a limit of detection (LOD) and limit of quantification (LOQ). They were calculated as signal-to-noise (S/N) ratios of 3 and 10, respectively, which were measured by using Analyst 1.6 software. The LODs of the analytical method for 13 mycotoxins in 5 types of cereal grains were between 0.1 and 18.1 ng/g, whereas the LOQs of the method for the mycotoxins in cereal grains were between 0.4 and 54.8 ng/g (Table S1). They were as low as those for detection of trace amounts of the toxins. It indicates that the method using LC/MS/MS is highly sensitive for determination of all 13 mycotoxins in cereal grains. 

### 2.5. Monitoring Levels of 13 Mycotoxins in Commercial Cereal Grains

The analytical method validated above was used for the determination of levels of 13 mycotoxins in 5 types of 507 cereal grains (brown rice, maize, millet, sorghum, and mixed cereal) collected from local markets in South Korea. The occurrence and levels of all 13 toxins in the commercial products are summarized in Table 1. Mycotoxins produced from *Fusarium* sp. (FB_1_, FB_2_, DON, NIV, and ZEN) were more frequently detected in all grain samples than other toxins such as AFs and OTA from other fungal species. 

The AFB_1_ showed 1%–9% of incidence in all cereal grains (Table 1). The highest levels of AFB_1_ in maize and millet were 5.2 and 5.6 ng/g, respectively, which were below the maximum allowable limit (10 ng/g) set by the Korean Food and Drug Administration (KFDA) [18]. However, levels of AFB_1_ in mixed cereal (12.4 ng/g) exceeded the allowable limit. The occurrence of AFB_2_ was much lower than that of AFB_1_; the occurrence of AFB_2_ was below 1% in millet. AFG_1_ and AFG_2_ were not detected in any types of grain samples.

The incidence (42%–95%) and levels of FB_1_ and FB_2_ were similar to each other in the same grain group, and the levels of Fs were in the range of 1.6–2990 ng/g in positive samples (Table 1). In particular, Fs were detected in relatively high concentrations (1.9–2990 ng/g of the range and 42.8–160.8 ng/g of the mean levels in positive samples) in maize and sorghum compared to those (1.6–80.1 ng/g of the range and 9.6–17.3 ng/g of the mean levels in positive samples) in other types of cereal grains. Nevertheless, the levels of FB_1_ and FB_2_ detected in all types of grains were below the maximum allowable limit (4 µg/g for FB_1_ and FB_2_ in maize) set by the KFDA.

Of trichothecenes (TCs), DON and NIV were more frequently detected in all types of cereal grains than T-2, HT-2, and 3-AcDON; the incidence of DON and NIV was between 16%–70% in all grains except 7% for DON and 5% for NIV in brown rice, and the mean levels of DON and NIV were in the range of 6.0-1405 ng/g in positive samples (Table 1). Except the levels of DON in maize, the levels of DON and NIV detected in all types of cereal grains were below the maximum allowable limit (1 µg/g for DON in grains and their products) set by the KFDA when the limit of DON is used for levels of NIV since the legal limits of NIV are not set yet in South Korea [18]. However, the occurrence of T-2, HT-2, and 3-AcDON was less than 2% in the cereal grains, and the ranges of those in positive samples were lower than 27.7 ng/g. The ranges of T-2, HT-2, and 3-AcDON were far below than 1 µg/g, which was set for DON as the legal limit by KFDA [18]. Thus, the levels of DON and NIV as well as those of 3AcDON, T-2, and HT-2 in all types of cereal grains except for DON in maize could not pose a health risk in South Korea. 

The incidence of ZEN, one of mycotoxins from *Fusarium* sp., was between 7%-62% in all types of cereal grains, and its range was between 0.2–313.0 ng/g (Table 1). In particular, the levels of ZEN detected in sorghum were above the maximum allowable limit (200 ng/g) set by the KFDA [18]. Therefore, the level of ZEN in sorghum could pose a risk to public health in South Korea.

Finally, OTA was detected rarely (0%–1% of incidence) in the cereal grains, and its range was 0–0.5 ng/g. The levels of OTA detected in all grains were below the maximum allowable limit (5 ng/g) set by the KFDA [18]. 

**Figure 4:**
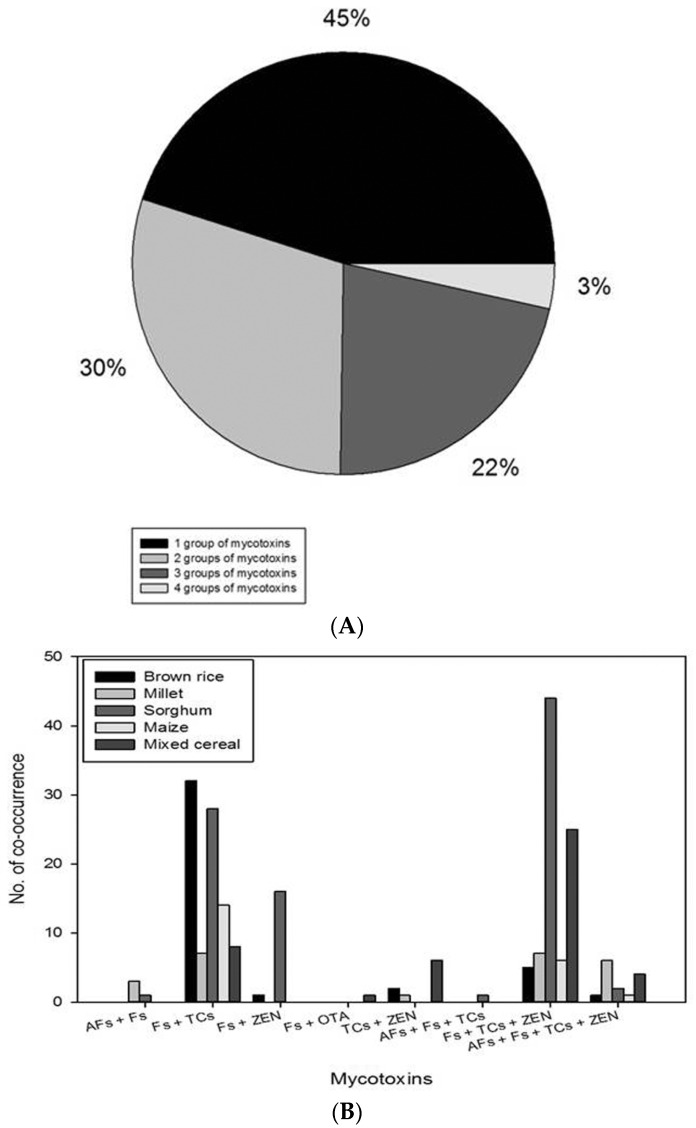
Co-occurrence of mycotoxins in the same sample of 5 types of cereal grains collected from retail market. (**A**) Percentage of co-occurrence of mycotoxins in a total of 507 cereal grains; (**B**) Number of co-occurrence of mycotoxins in 5 types of cereal grains.

The co-occurrence of the mycotoxins in 507 cereal grains was as follows; 30% of 2 groups of micotoxins, 22% of 3 groups of mycotoxins, and 3% of 4 groups of mycotoxins as shown in Figure 4A. In samples which showed co-occurrence of 2 groups of mycotoxins, Fs and TCs were frequently detected. Fs, TCs, and ZEN were major mycotoxins in the category of 3 groups of mycotoxins. These data on the co-occurrence of the mycotoxins in the same grain sample indicate that mycotoxins produced from *Fusarium* sp. (FB_1_, FB_2_, HT-2, T-2, DON, NIV, 3-AcDON, and ZEN) were concurrently detected in the same samples. In addition, they were detected more frequently along with AFB_1_ or AFB_2_ in the same samples than along with OTA, and any samples were not co-contaminated with AFs and OTA; AFs with *Fusarium* mycotoxins in 17 samples and OTA with *Fusarium* mycotoxins in only 1 mixed cereal sample (Figure 4B).

Overall, although the levels of AFB_1_ (except for mixed cereal)_,_ total AFs, FB_1_, FB_2_, and OTA detected in all 5 types of grain samples were below the maximum allowable limit set by the KFDA, they should have been much lower than observed levels because even low exposure to dietary toxins could pose a carcinogenic risk to human. In addition, the levels of DON and NIV in all 5 types of grains (except the levels of DON in maize) as well as those of 3AcDON, T-2, and HT-2 in the cereal grains were below the maximum allowable limit set for DON by KFDA. Also, the level of ZEN detected in sorghum was above the maximum allowable limit set by KFDA. Therefore, monitoring the levels of 13 mycotoxins in cereal grains marketed in South Korea should be continued.

## 3. Discussion

In this study we developed an improved rapid one-step method to co-elute all 13 major mycotoxins from Myco6in1 with IAC using 5 mL of 80% MeOH containing 0.5% acetic acid and established an analytical method for simultaneous determination of the mycotoxins by LC/MS/MS. The method was then used for rapid and simultaneous determination of levels of the 13 mycotoxins in grains collected from retail markets in South Korea. For 100% elution of 13 mycotoxins from IACs, all bonds between analytes and antibodies should be broken. In our one-step elution, 5 mL of 80% MeOH can provide enough time to break the bonds, and the acidic condition produced by 0.5% acetic acid makes the bond breakage occur easily, which improves the recoveries of the mycotoxins in this study. The recovery rates for 13 mycotoxins in 5 types of matrices were 73.9%–133.0% using the improved toxin extraction method (Figure 3). In contrast, a previous study described by Lattanzio and collaborators showed that recovery rates were relatively low (63%–93%) in simultaneous determination of multi-mycotoxins in corn and wheat samples [14].

Our data showed that the incidence of AFB_1_ in all cereal grains was from 1% to 9% (Table 1). Of these grains, the occurrence of AFB_1_ in maize was 1% in our study, while AFB_1_ was not detected in corn collected in South Korea in one study described by Park and co-workers [19]. One of the reasons for this discrepancy may have come from the small sample size (*n* = 18) in the previous study.

Also, the occurrences of FB_1_ and FB_2_ in brown rice and millet were about 50% (42% in brown rice and 52% in millet for FB_1_, and 44% in brown rice and 50% in millet for FB_2_) in the current study (Table 1), while both mycotoxins were not detected in brown rice and millet collected in South Korea in one study described by Seo and collaborators [20]. Again, the small sample size (*n* = 12) in the previous study may have been one of the reasons for this discrepancy.

Table 1 showed that the incidence and the mean levels of DON and NIV in maize were similar to each other (13% and 180 ng/g for DON, and 18% and 116 ng/g for NIV). These results show lower occurrences and levels of DON and NIV compared to those detected in corn collected in South Korea in 1993, in which authors reported occurrences of 65.2% of DON (310 ng/g of the mean level) and 34.8% of NIV (77 ng/g of the mean level) [21]. One of possible reasons for this discrepancy may have been due to different climate when the maize was harvested in the field or differences between the regions where the maize was harvested. 

The incidence and the mean level of ZEN, another mycotoxin from *Fusarium* sp., in maize were 7% and 4.3 ng/g in the present study (Table 1). The level of ZEN in maize was lower than that (151 ng/g of the mean level) detected in corn collected in South Korea in 1993 as described by Kim and co-workers, while the occurrence of ZEN in maize in our study was similar to that (8%) detected in the previous study [21]. The same reason (different climate when maize was harvested or different regions where maize was harvested) as that for DON, and NIV may be able to explain this discrepancy.

Finally, OTA was detected rarely (1% of incidence) in all cereal grains, and its mean level was 0–0.5 ng/g in our study (Table 1). Of these grains, OTA was not detected in maize in this study, which was the same as that (0%) in corn collected in South Korea in 2002 [19].

On the other hand, Figure 4B showed that 17 samples were co-contaminated with AFs and mycotoxins from *Fusarium* sp. (FB_1_, FB_2_, HT-2, T-2, DON, NIV, 3-AcDON, and ZEN), whereas only one sample (mixed cereal) was concurrently contaminated with OTA and *Fusarium* mycotoxins. These data are in agreement with Ok and co-workers’ study in which they detected DON and ZEN concurrently in 12 out of 70 corn and barley samples collected from South Korea in 2005 and 2006 [22]. In addition, another study from South Korea reported the co-occurrence of AFB_1_, FB_1_, and ZEN in corn and barley [19]. Moreover, studies from other countries also showed the co-occurrence of the mycotoxins from *Fusarium* sp. One study from Finland reported that the same samples of cereal grains such as barley, oats, and wheat were contaminated simultaneously with DON, 3-AcDON, HT-2, T-2, and ZEN [23]. Another study by Ali and collaborators also reported that Fs, DON, NIV, and ZEN were co-contaminated with AFs in corn from Indonesia, and they isolated *Aspergillus flavus* and *Aspergillus parasiticus* along with *Fusarium moniliforme* in the same samples [24]. These data suggest that our cereal samples may also have been co-infected with the *Aspergillus* sp. and *Fusarium* sp. 

Because the co-occurrence of these mycotoxins is common in cereal grains and they can cause synergistic effects to human and animals, it is necessary that efficient control methods are developed to prevent and monitor contamination of multi-mycotoxins and fungi in grains. 

## 4. Conclusions

In our current study we developed a highly sensitive and reliable analytical method for simultaneous determination of levels of 13 mycotoxins (AFB_1_, AFB_2_, AFG_1_, AFG_2_, DON, NIV, 3-AcDON, FB_1_, FB_2_, T-2, HT-2, ZEN, and OTA) in cereal grains by LC/MS/MS after IAC clean-up. We were able to minimize any interfering materials against determination of levels of the mycotoxins in grains using the Myco6in1 with IAC columns and were able to elute all of the mycotoxins simultaneously from the IAC using 5 mL of 80% MeOH containing 0.5% acetic acid. The analytical method established in this study showed a good linearity, sensitivity, specificity, and accuracy in determination of levels of the mycotoxins by LC/MS/MS. The recovery rates of the mycotoxins in rice were 73.9%–133.0% along with RSD_r_ of 0.1%–14.3%, which satisfied the legal limits of the recovery and RSD recommended by Codex or AOAC. The LODs of the analytical method for all of the mycotoxins were in the range of 0.1–18.5 µg/kg at a signal-to-noise (S/N) ratio of 3, and the LOQs of the method for the mycotoxins were in the range of 0.4–56.1 µg/kg at an S/N ratio of 10.

Finally, we investigated levels of 13 mycotoxins in 5 types of commercial cereal grains (brown rice, maize, millet, sorghum, and mixed cereal) collected from local markets in South Korea using the analytical method established in this study. The levels of DON and NIV in all types of cereal grains (except levels of DON in maize) and those of 3-AcDON, T-2, and HT-2 in cereal grains were below the maximum allowable legal limit (1 µg/g) set for DON or NIV by the KFDA. The levels of AFB_1_ (except for mixed cereal)_,_ total AFs, FB_1_, FB_2_, ZEN (except for sorghum) and OTA in the cereal grains were also below the maximum allowable limit set by the KFDA in South Korea. Because levels of DON in maize, those of AFB_1_ in mixed cereal, and those of ZEN in sorghum were higher than the maximum legal limits set by KFDA, extensive and active research should be continued for monitoring all 13 mycotoxins in cereal grains. Furthermore, establishment of legal limits of trichothecenes including NIV, 3-AcDON, T-2, and HT-2 for grains marketed in South Korea is required for monitoring them.

## 5. Experimental Sections

### 5.1. Samples

Brown rice, millet, sorghum, maize, and mixed cereal were used for the development of the analytical method and the determination of levels of 13 mycotoxins in these grains. The 5 types of 507 cereal grains were purchased from retail markets in South Korea. The grain samples were stored at 4 °C until use.

### 5.2. Standard Solutions and Reagents

The standards of Aflatoxins Mix 1 (2 µg/mL for AFB_1_ and AFG_1_, and 0.5 µg/mL for AFB_2_ and AFG_2_), OTA (10 µg/mL), Fumonisin Mix 3 (50 µg/mL for FB_1_ and FB_2_), DON (100 µg/mL), NIV (100 µg/mL), and 3-AcDON (100 µg/mL) were obtained from Biopure (Cambridge, MA, USA), while those of ZEN (100 µg/mL), T-2 (100 µg/mL), and HT-2 (100 µg/mL) were obtained from Sigma-Aldrich (St. Louis, MO, USA). The standard of 3 TCs mixture (DON, NIV, and 3-AcDON) was prepared by mixing 100 µL of each TC (101.9 µg/mL of DON, 100.9 µg/mL of NIV, and 100.6 µg/mL of 3-AcDON, which were diluted with acetonitrile [ACN]) with 490 µL of ACN, followed by dilution of the mixture with 410 µL of MeOH. The phosphate buffered saline (PBS, pH 7.4) for pH adjustment of toxin extracts was purchased from Sigma-Aldrich (St. Louis, MO, USA). HPLC grade ACN and MeOH were obtained from Merck (Darmstart, Germany). Stock solutions for each toxin were prepared by dilution of the standard solutions with 100% ACN (except for FB_1_ and FB_2_ for which 50% ACN was used), and working solutions with a series of toxin concentrations were made by dilution with 50% MeOH containing 1% acetic acid. 

### 5.3. Assessment of the Precision, Linearity, and Sensitivity of the Analytical Method forDetermination ofLevels of 13 Mycotoxins

The linearity of a series of concentrations of 13 toxins in the analytical method was assessed by each standard curve using five levels (1.325, 2.65, 13.25, 26.5, and 53 ng/mL) of standard solutions for each toxin. The calibration curve for each toxin was constructed by plotting the peak areas (*y* axis) versus concentrations of each toxin (*x* axis) in LC/MS/MS analyses (Figure S1).

Five types of cereal grains that were not naturally contaminated with toxins were spiked with a mixed standard solution including 13 mycotoxins to determine the precision of the analytical method at levels of the toxins as follows: NIV, 321.0 ng/g; DON, 326.1 ng/g; 3-AcDON, 321.9 ng/g; AFB_1_, 8.5 ng/g; AFB_2_, 8.5 ng/g; AFG_1_, 8.2 ng/g; AFG_2_, 8.0 ng/g; FB_1_, 163.2 ng/g; FB_2_, 160.3 ng/g; HT-2, 161.6 ng/g, T-2, 64.6 ng/g; ZEN, 64.3 ng/g; OTA, 16.1 ng/g. Extraction and clean-up of analytes from the spiked samples were performed in triplicate by the procedures described below.

The sensitivity of the analytical method using LC/MS/MS was determined by LOD and LOQ. They were calculated as signal-to-noise (S/N) ratios of 3 and 10, respectively, which were determined by using HPLC software (Analyst 1.6 software program, Sciex, Framingham, MA, USA). 

### 5.4. Toxin Extraction Procedure

Each sample (12.5 g) was weighed and placed in 100 mL of Erlenmeyer flasks after being ground into powders by a food grinder (Hallde, KISTA, Sweden). Fifty milliliters of ACN containing acetic acid (ACN: water: acetic acid = 79.5:20.0:0.5; v:v:v) as a selected solvent was added to it, and the 13 toxins were extracted by shaking at 320 rpm for 1 h with a wrist action shaker (EYELA, Tokyo, Japan). After the extracts were centrifuged at 3000× *g* for 5 min under 4 °C, supernatants were filtered through Whatman No. 4 filter paper (Whatman^TM^, Maidstone, UK). Five milliliters of each filtrate was diluted with 75 mL of PBS (pH 7.4) and then filtered through a GF/A filter paper (Whatman^TM^, Maidstone, UK). Sixty-five milliliters of the filtrate was loaded onto an IAC (Myco6in1+ column, VICAM, Milford, MA, USA) and passed through at a flow rate of one—two drops/sec. The column was washed with 10 ml of PBS and distilled water until 2–3 mL of air passed through it, and toxins were finally eluted from the column with 5 mL of 80% MeOH containing 0.5% acetic acid. The eluates were evaporated to dryness under a stream of N_2_ at 50 °C using a vacuum manifold (Agilent, Santa Clara, CA, USA), and the residues were re-dissolved in 1 mL of 50% MeOH containing 0.5% acetic acid. The solutions were vortexed for 1 min and filtered through a 0.22 μm syringe filter. 

### 5.5. Matrix Effects on Toxin Extraction

After extraction of matrix components from the 5 types of cereal grains by the same procedure as the toxin extraction method described above, 265 ng/mL of each toxin standard solution was mixed with the extract from each type of grain sample at a ratio (*v*:*v*) of 4 to 1 to make 53 ng/mL of each toxin standard solution containing the extracted matrix components. Then, a series of five levels (1.325, 2.65, 13.25, 26.5, and 53 ng/mL) of standard solutions for each toxin were prepared by serial dilution of 53 ng/mL of the standard solution with the extract containing matrix components. After LC/MS/MS analyses, each calibration curve for each toxin was constructed as described above. The matrix-matched calibration curves were used for calculation of recovery rates of 13 mycotoxins from 5 types of matrices.

### 5.6. LC/MS/MS Conditions

HPLC (1260 series, Agilent, Santa Clara, CA, USA) equipped with a AB SCIEX QTRAP mass spectrometer (AB 3200, Applied Biosystems, Foster city, CA, USA) was used to detect the 13 toxins. Separation of the toxins was carried out on a Scherzo SM-C18 column (3 mm × 150 mm, 3 µm particle size; Imtakt, Kyoto, Japan). The two elution solutions used were (A) 0.1% formic acid in water containing 2 mM ammonium acetate and (B) 0.1% formic acid in MeOH containing 2 mM ammonium acetate. The solutions were pumped at a flow rate of 0.5 mL/min and a gradient elution program was applied as shown in Table 2. The injection volume of the samples was 10 μL. Analysis software (version 1.6, Sciex, Framingham, MA, USA) was used to control the LC/MS/MS system and to acquire and process data. The mass spectrometer was operated in the positive ESI (electrospray ionization) mode with MRM (multiple reaction monitoring) at unit resolution. The main MS parameters were optimized and finally set as follows: curtain gas, 20 psi; collision gas (CAD), medium; capillary temperature (TEM), 500 °C; ion spray voltage, ± 4500 V; ion source gas 1 (GS1), 50 psi; ion source gas 2 (GS2), 50 psi; interface heater (ihe), on. Nitrogen was used as the nebulizer, heater, curtain, and collision gas.

MRM parameters for detection of 13 mycotoxins by the mass spectrometer are shown in Table 3. 

## Figures and Tables

**Table 1:** Mean and range of levels and incidence of 13 mycotoxins in a total of 507 brown rice, millet, sorghum, maize, and mixed cereal collected from retail market.

Mycotoxin^1^		Grain Sample				
	Item	Brown Rice	Millet	Sorghum	Maize	Mixed Cereal
AFB_1_	Incidence (%)	1	9	4	1	4
	Mean^2^ (ng/g)	1.1	1.3	1.0	5.2	4.3
	Range (ng/g)	1.1	0.4–5.6	0.7–1.7	5.2	0.7–12.4
AFB_2_	Incidence (%)	0	1	0	0	0
	Mean^2^ (ng/g)	0	0.5	0	0	0
	Range (ng/g)	-	0.5	-	-	-
FB_1_	Incidence (%)	42	52	95	47	74
	Mean^2^ (ng/g)	13.6	12.4	160.8	136.5	17.3
	Range (ng/g)	2.1–22.8	2.0–32.6	5.8–890.0	3.8–2990.0	3.1–80.1
FB_2_	Incidence (%)	44	50	89	59	58
	Mean^2^ (ng/g)	9.6	12.0	42.8	45.2	14.6
	Range (ng/g)	1.6–18.8	1.6–31.1	4.0–223.5	1.9–620.0	1.8–22.1
T-2	Incidence (%)	0	0	0	2	0
	Mean^2^ (ng/g)	0	0	0	10.0	0
	Range (ng/g)	-	-	-	6.4–13.7	-
HT-2	Incidence (%)	0	0	0	0	1
	Mean^2^ (ng/g)	0	0	0	0	4.3
	Range (ng/g)	-	-	-	-	4.3
OTA	Incidence (%)	0	0	0	0	1
	Mean^2^ (ng/g)	0	0	0	0	0.5
	Range (ng/g)	-	-	-	-	0.5
DON	Incidence (%)	7	25	70	13	19
	Mean^2^ (ng/g)	5.6	46.5	64.0	180.4	41.5
	Range (ng/g)	6.0–12.3	12.1–212.0	18.1–257.0	17.0–1405.0	14.5–162.0
NIV	Incidence (%)	5	16	53	18	40
	Mean^2^ (ng/g)	26.3	45.3	48.2	116.1	50.2
	Range (ng/g)	16.3–36.8	15.7–102.0	18.1–211.5	12.7–570.0	13.8–175.0
3-AcDON	Incidence (%)	0	0	0	2	0
	Mean^2^ (ng/g)	0	0	0	17.8	0
	Range (ng/g)	-	-	-	7.9–27.7	-
ZEN	Incidence (%)	32	14	62	7	47
	Mean^2^ (ng/g)	5.2	7.4	37.5	4.3	6.1
	Range (ng/g)	0.4–37.6	0.7–61.5	0.9–313.0	0.9–14.7	0.2–36.0

^1^ AFG_1_ and AFG_2_ were not detected in all grain samples; ^2^ Mean indicates an average in positive samples.

**Table 2:** A gradient condition of a mobile phase composed of two solutions in analyses of multi-mycotoxins by HPLC.

Total Time (min)	Flow Rate (mL/min)	A Solution (%)	B Solution (%)
0	0.5	70	30
3	0.5	70	30
13	0.5	10	90
16	0.5	10	90
18	0.5	70	30
20	0.5	70	30

**Table 3:** Multiple reaction monitoring (MRM) parameters for detection of 13 mycotoxins by the mass spectrometer.

Mycotoxin	Precursor ion	Q1 (*m/z*)	Q3 (*m/z*)	Time (msec)	DP (volts)	EP (volts)	CE (volts)	CXP (volts)
NIV	[M + CH_3_COO^−^]^−^	313 313	175 295	100 100	41 41	5 5	19 15	6 6
DON	[M + CH_3_COO^−^]^−^	297.2 297.2	249.2 231.2	100 100	40 40	7.5 7.5	12 12	4 4
3-AcDON	[M + CH_3_COO^−^]^−^	339 339	231 213	100 100	15 15	3.5 3.5	15 15	2 2
AFB_1_	[M + H]^+^	313 313	285.1 115	100 100	70 70	11 11	51 89	4 4
AFB_2_	[M + H]^+^	313 313	287.1 259.1	100 100	70 70	10 10	50 50	3 3
AFG_1_	[M + H]^+^	329 329	243.0 215.1	100 100	70 70	11 10	25 50	4 4
AFG_2_	[M + H]^+^	331 331	189 257	100 100	70 70	10.0 10.5	50 47	3 4
FB_1_	[M + H]^+^	722.4 722.4	334.3 352.4	100 100	91 91	8 8	53 43	6 6
FB_2_	[M + H]^+^	706.4 706.4	336.4 318.2	100 100	70 70	9 9	45 47	6 6
HT-2	[M + NH_4_]^+^	442 442	263 215	100 100	26 26	3 3	19 19	4 4
T-2	[M + NH_4_]^+^	484.1 484.1	215.2 185.2	100 100	21 21	6 6	29 31	4 4
ZEN	[M − H]^−^	317.1 317.1	131 175	100 100	−50 −50	−4.5 −4.5	−40 −34	−2 −2
OTA	[M + H]^+^	404 404	239 220.9	100 100	41 41	5 5	31 19	4 4

## Supplementary Materials

The following are available online at www.mdpi.com/2072-6651/9/3/106/s1, Figure S1: Calibration curves of 13 mycotoxins, Table S1: LODs and LOQs of 13 mycotoxins in 5 types of cereal grains.

## Abbreviations

The following abbreviations are used in this manuscript:

LC/MS/MS	Liquid Chromatography-Tandem Mass Spectrometry
AFs	aflatoxins
DON	deoxynivalenol
3-AcDON	3-Acetyldeoxynivalenol
OTA	ochratoxin A
Fs	fumonisins
NIV	nivalenol
ZEN	zearalenone
T-2	T-2 toxin
HT-2	HT-2 toxin
TCs	Trichothecenes
TIC	Total Ion Chromatogram
EIC	Extract Ion Chromatogram
RSD_r_	relative standard deviation
LOD	Limit of Detection
LOQ	Limit of Quantification
ACN	acetonitrile
MeOH	methanol
MRM	multiple reaction monitoring
