# Supplementary material for: Simultaneous Determination of Multi-Mycotoxins in Cereal Grains Collected from South Korea by LC/MS/MS
Source: Toxins (Basel). 2017 Mar 16;9(3):106. doi: 10.3390/toxins9030106 (PMC5371861; doi:10.3390/toxins9030106)
Supplement: Supplementary file 1 [file toxins-09-00106-s001.pdf]

Supplementary Materials: Simultaneous Determination of Multi-Mycotoxins in Cereal Grains Collected from South Korea by LC/MS/MS

Dong-Ho Kim, Sung-Yong Hong, Jea Woo Kang, Sung Min Cho, Kyu Ri Lee, Tae Kyung An, Chan Lee and Soo Hyun Chung

(A)

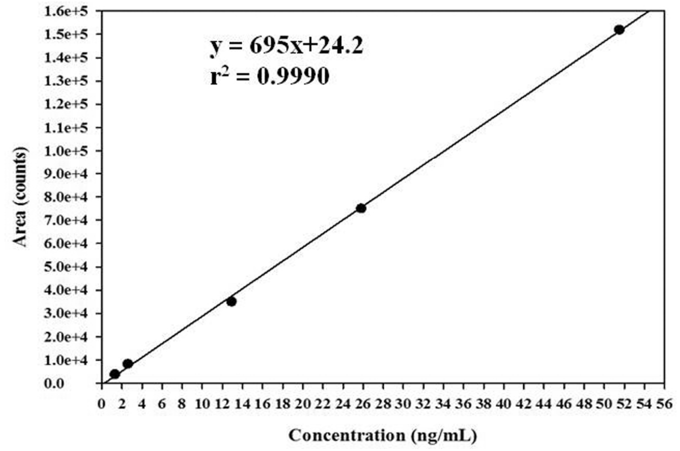

(B)

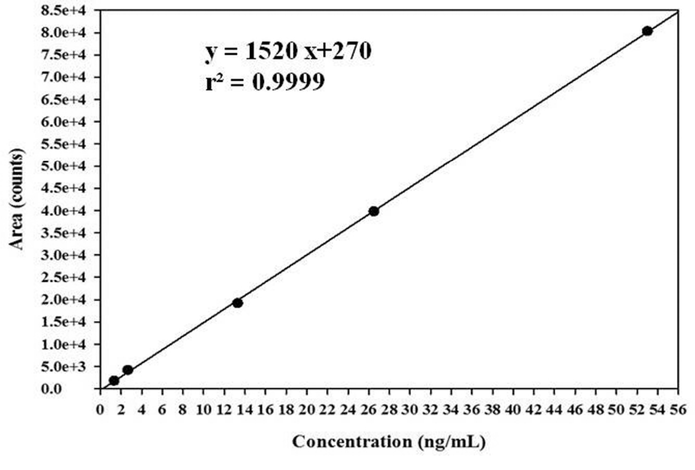

(C)

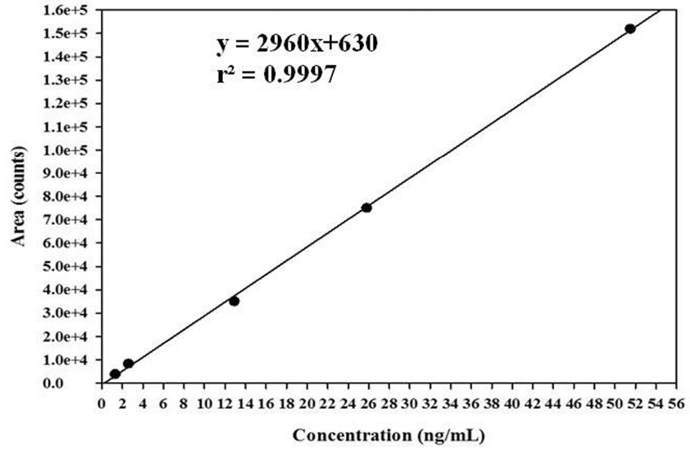
y = 2960x+630
r² = 0.9997
Area (counts)
Concentration (ng/mL)

(D)

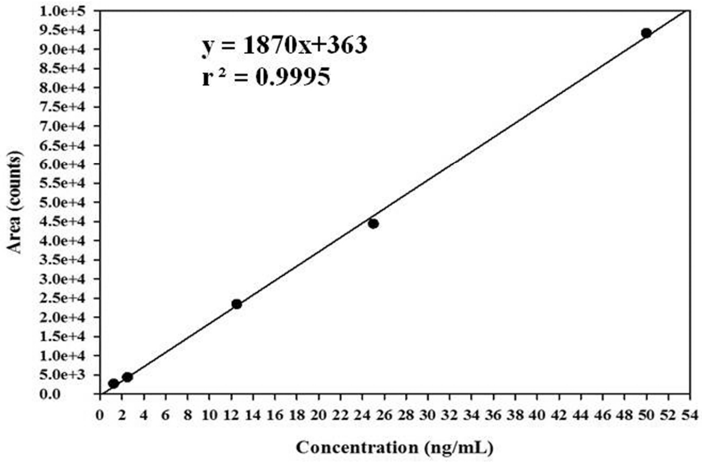
y = 1870x+363
r ² = 0.9995
Area (counts)
Concentration (ng/mL)

(E)

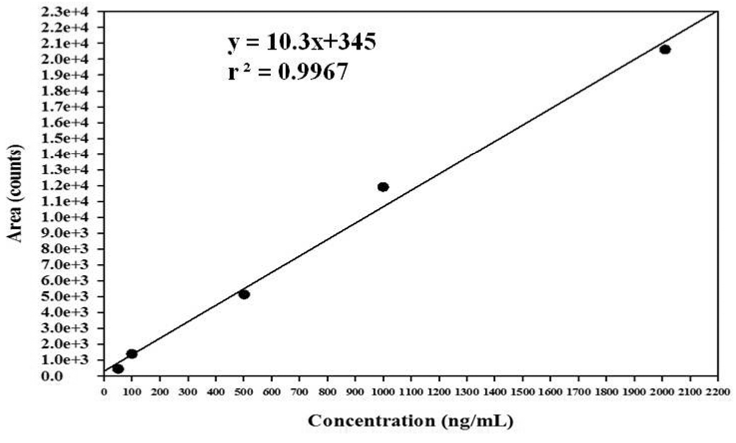
y = 10.3x+345
r ² = 0.9967
Area (counts)
Concentration (ng/mL)

(F)

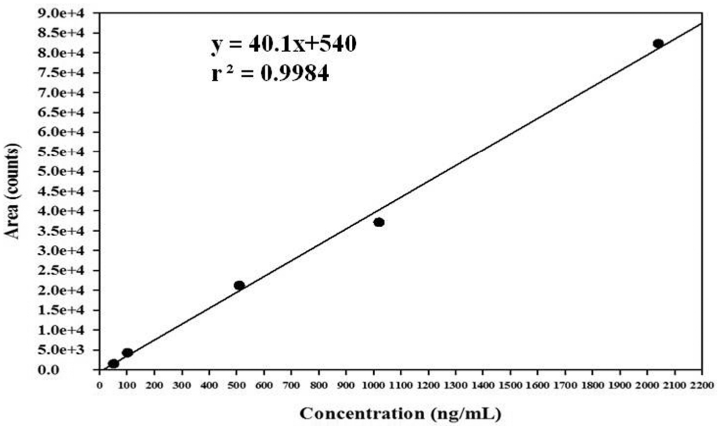
y = 40.1x+540
r ² = 0.9984
Area (counts)
Concentration (ng/mL)

(G)

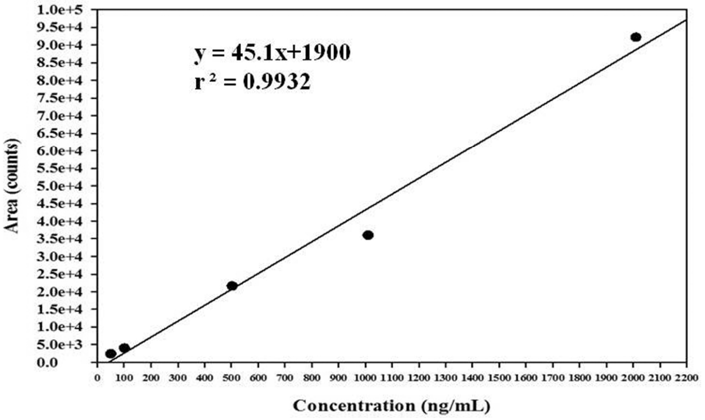
y = 45.1x+1900
r ² = 0.9932
Area (counts)
Concentration (ng/mL)

(H)

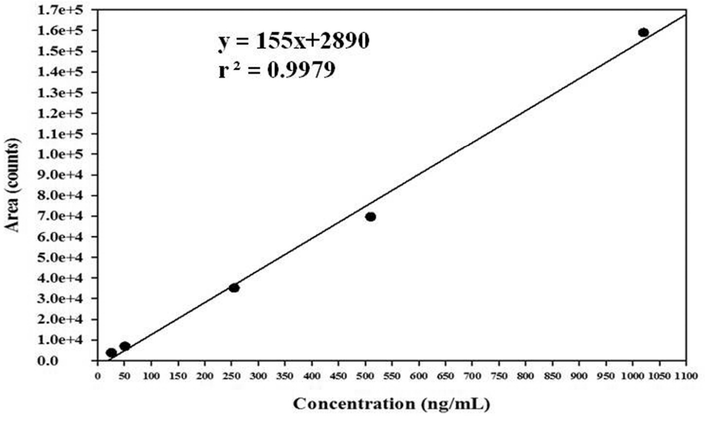
y = 155x+2890
r ² = 0.9979
Area (counts)
Concentration (ng/mL)

(I)

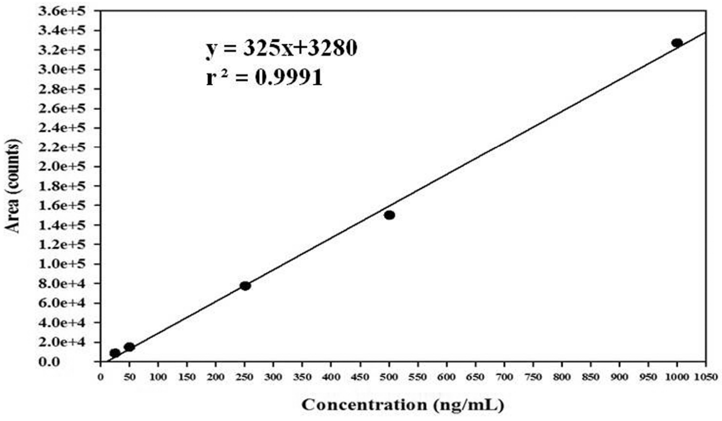
y = 325x+3280
r ² = 0.9991
Area (counts)
Concentration (ng/mL)

(**i**)

(J)

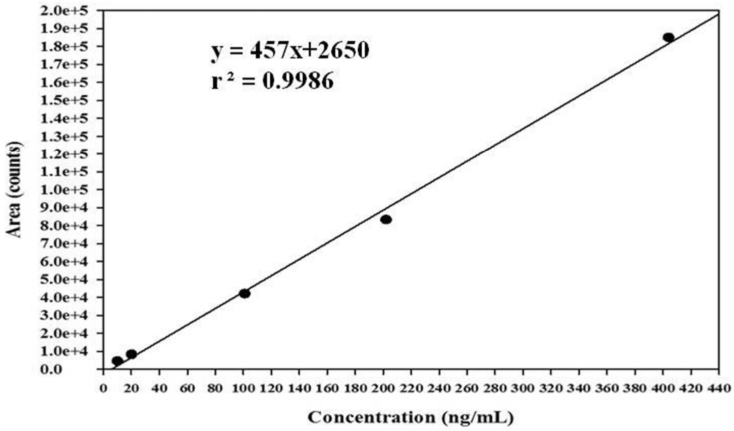
y = 457x+2650
r ² = 0.9986
Area (counts)
Concentration (ng/mL)

(K)

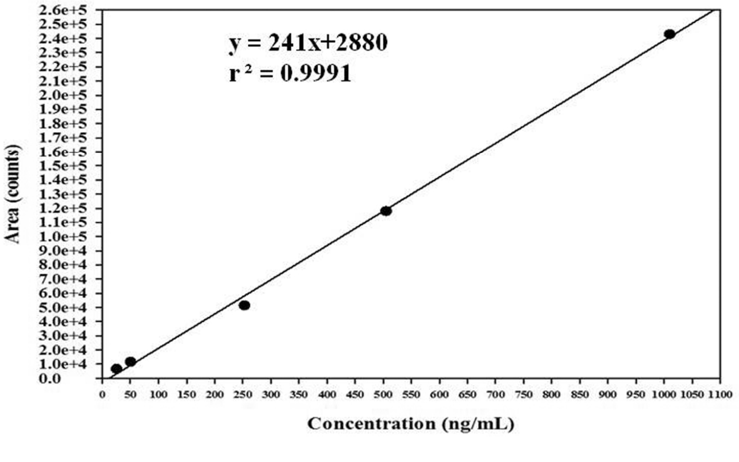
y = 241x+2880
r ² = 0.9991
Area (counts)
Concentration (ng/mL)

(L)

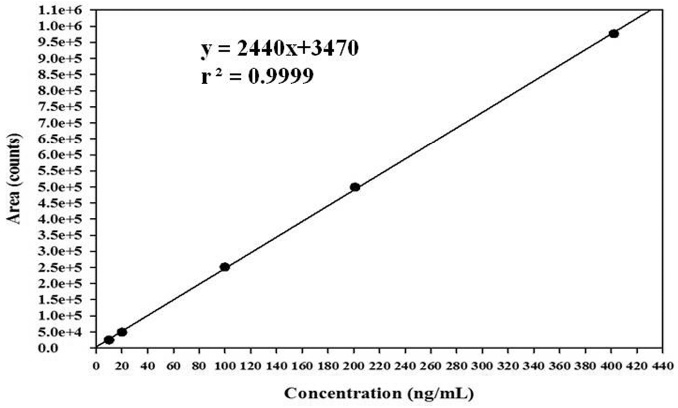

(M)

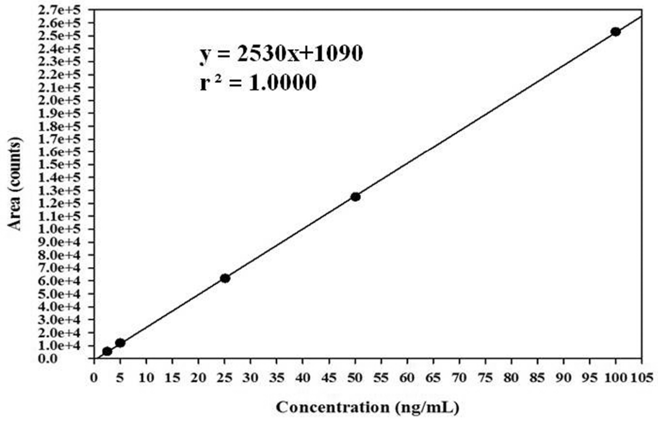

Figure S1. Calibration curves of 13 mycotoxins. A series of standard solutions for each toxin were prepared in the range of 1.325, 2.65, 13.25, 26.5, and 53 ng/mL. The standard solutions for each toxin were injected into LC/MS/MS in triplicate. Calibration curves of (**A**) AFB_1 (**B**) AFB_2 (**C**) AFG_1 (**D**) AFG_2 (**E**) NIV (**F**) DON (**G**) 3-AcDON (**H**) FB_1 (**I**) FB_2 (**J**) T-2 (**K**) HT-2 (**L**) ZEN (**M**) OTA are shown.

Table S1. LODs and LOQs of 13 mycotoxins in 5 types of cereal grains.

Cereal grain	Toxin	LOD (S/N=3, ng/g)	LOQ (S/N=10, ng/g)	Toxin	LOD (S/N=3, ng/g)	LOQ (S/N=10, ng/g)
Brown rice	NIV	14.9	45.1	DON	6.0	18.3
Millet		15.2	46.1		12.1	36.7
Sorghum		18.1	54.8		17.7	53.6
Maize		12.4	37.6		17.0	51.4
Mixed cereal		13.8	41.8		12.6	38.2
Brown rice	3-AcDON	2.5	7.6	ZEN	0.4	1.3
Millet		4.8	14.5		0.7	2.0
Sorghum		4.5	13.6		0.5	1.5
Maize		4.7	14.2		0.8	2.5
Mixed cereal		4.7	4.7		0.2	0.6
Brown rice	AFB_1	0.3	0.8	AFB_2	0.1	0.4
Millet		0.4	1.1		0.3	0.8
Sorghum		0.7	2.2		0.4	1.1
Maize		0.7	2.1		0.5	1.4
Mixed cereal		0.4	1.2		0.2	1.0
Brown rice	AFG_1	1.1	3.4	AFG_2	0.1	0.4
Millet		1.0	3.0		0.3	0.9
Sorghum		1.6	4.8		0.5	1.4
Maize		0.8	2.3		0.6	1.9
Mixed cereal		0.1	0.4		0.3	1.0
Brown rice	FB_1	0.8	2.3	FB_2	0.7	2.2
Millet		1.8	5.4		1.4	4.4
Sorghum		1.5	4.4		3.5	10.6
Maize		2.4	7.3		1.9	5.9
Mixed cereal		0.9	2.6		1.7	5.3
Brown rice	HT-2	12.6	38.3	T-2	1.9	5.8
Millet		15.3	46.4		2.5	7.5
Sorghum		15.3	46.4		4.0	12.0
Maize		11.6	35.1		6.3	19.2
Mixed cereal		4.0	12.2		3.1	9.3
Brown rice	OTA	0.6	1.9			
Millet		2.3	7.0			
Sorghum		2.3	7.0			
Maize		1.8	5.4			
Mixed cereal		0.4	1.4			
